# Fluorescent analyses of sediments and near-seabed water in the area of the WW2 shipwreck “Stuttgart”

**DOI:** 10.1038/s41598-024-75279-3

**Published:** 2024-10-19

**Authors:** Emilia Baszanowska, Zbigniew Otremba, Maria Kubacka

**Affiliations:** 1 Department of Physics, Gdynia Maritime University, 81-225 Gdynia, Poland; 2 Department of Operational Oceanography, Maritime Institute, Gdynia Maritime University, ul. Roberta de Plelo 20, 80-848 Gdańsk, Poland

**Keywords:** Ecology, Environmental sciences, Ocean sciences

## Abstract

Motorship wrecks on the seabed pose a serious threat to the marine environment due to oil leaking from their fuel tanks. Such substances can penetrate the sediments and enter the water. There is a need to analyse bottom water and seabed sediment samples for the content of toxic petroleum substances. Tests were undertaken on samples collected near ​​the site of the World War II shipwreck of the s/s “*Stuttgart*”. The wreck is located in the Baltic Sea, in the Gulf of Gdańsk. To answer whether toxic hydrocarbons from wrecks enter the sea environment, a fluorometric analysis was carried out based on measurements of excitation-emission ultraviolet spectra for sediments and near-seabed water. The results of these analyses indicate the presence of oil substances in the sediments and the bottom water at some sampling points close to the wreck site. Studies have shown that the applied method of the so-called fluorometric indicator allows for determining the sites of water pollution with oil substances hidden in sediments.

## Introduction

Oil spills at sea pose a serious threat to the natural marine ecosystem^1–3^. Therefore, the key to protecting the natural marine environment is prevention, early detection and combating of oil spills^4–6^. Potential sources of leaks at sea may be discharges from ships^7^, natural seeps from the bottom^8–10^, and tanker disasters, the number of which is increasing significantly due to the growing importance of maritime transport in economic development. Other major sources of oil spills at sea are ruptured pipelines (off Santa Barbara, California in 2015)^11^, drilling rigs/platforms (Deepwater Horizon 2010)^12^ or shipwrecks lying on the seabed^13–14^. Dangerous to the marine environment are potentially polluting wrecks (PPW), which are widely studied^15^.

The bottom of the seas and oceans hides a lot of wrecks, which may contain oily substances in their fuel tanks. Over 3,500 shipwrecks dating back to the Second World War (WW2) have been registered^16^. The bottom of the Baltic Sea also hides shipwrecks, munitions and chemical weapons^17^. History shows that there are 8–10 thousand wrecks in the Baltic Sea from various historical periods^18^. Some of the 20th-century wrecks may contain significant amounts of fuel^19–22^. Fuel present in wrecks may leak into the sea after some time. This poses a serious threat to the natural marine environment and public health due to the toxic properties of substances contained in oils.

**Fig. 1:**
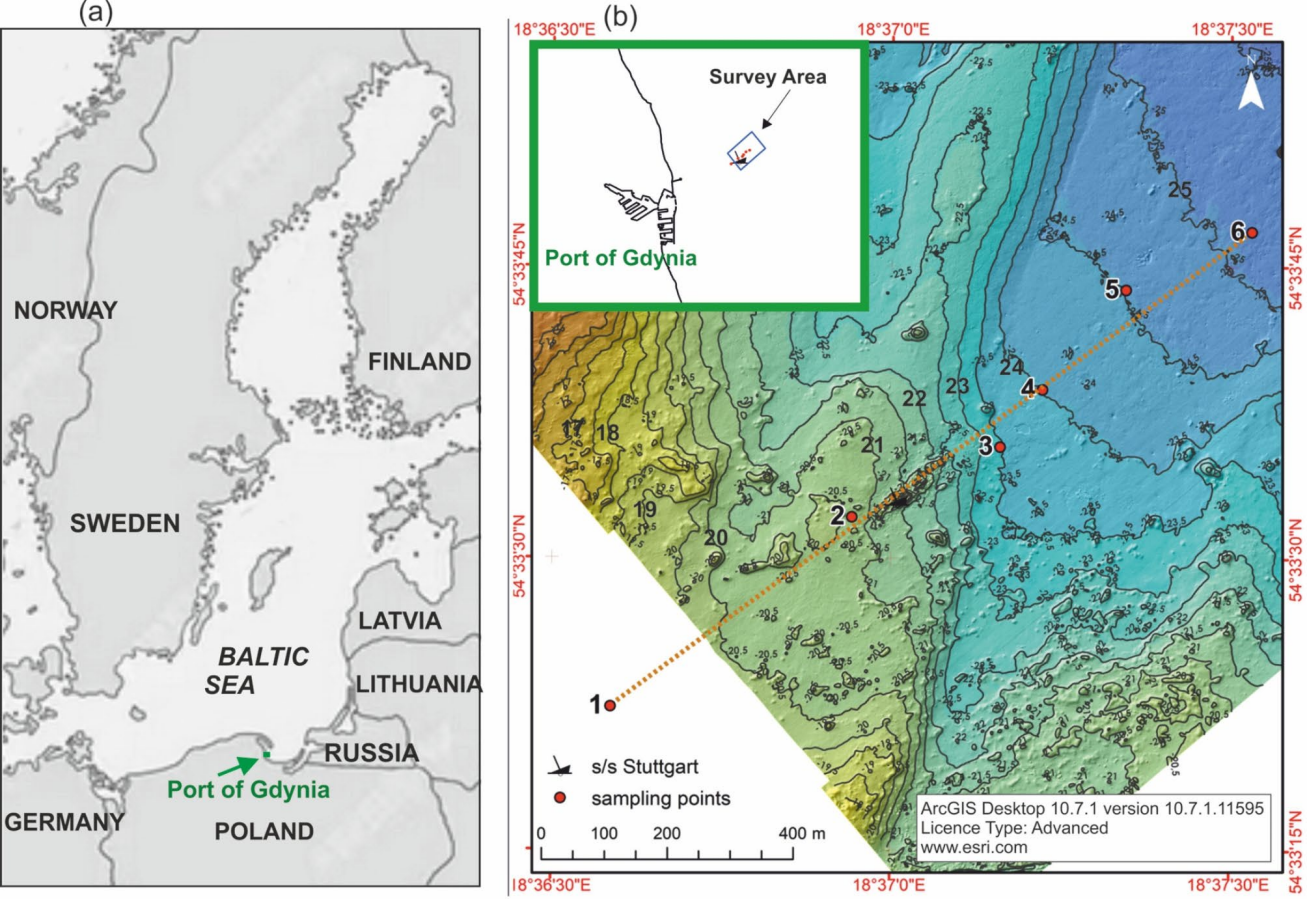
Location of the sampling points (**a**), a 3D projection of the seabed in the area of the *s/s Stuttgart* wreck (depth levels represented by specific colours) with marked sampling stations; bathymetric data source: Department of Operational Oceanography, Maritime Institute. Data collected using a Teledyne Reson SeaBat 7125 Multibeam Echosounder (**b**). Map created in ArcGIS version 10.7.1.11595, http://www.esri.com.

One of the places at risk of oil substances coming from a shipwreck is a part of the Gulf of Gdańsk near the Port of Gdynia (Fig. 1). Since 1943, it has been the location of the wreck of the deliberately sunk German ship *s/s Stuttgart*. After being bombed in the Port of Gdynia, the ship was on fire, so it was towed out of the port^23^. After the Second World War, the wreck, as a navigational obstacle, was partially destroyed using pyrotechnics. Now only fragments of the ship protrude above the seabed. According to scattered Internet sources, the original length of the ship was 171.6 m, and now the ship remains stretch over a distance of 167.8 m.

Oil substances are mixtures of carbon compounds that contain various types of hydrocarbons, including toxic polycyclic aromatic hydrocarbons (PAHs)^24–26^. PAHs are slightly soluble in water^27^. However, they have an affinity for colloids and sorbents^28–30^. This causes them to get into the sediments easily. Rogowska^23^ reports the enrichment of sediments near the wreck site with PAHs. Moreover, fuel present in shipwrecks favours the development of microorganisms, fungi and cyanobacteria^31^. The *s/s Stuttgart* wreck raises significant concerns due to its location at a shallow depth of approximately 20 m, within a coastal fishing area and near an urbanised region with beaches attractive to tourists. Additionally, this site is a critical waterway for commercial shipping, situated along heavily trafficked sea routes. Oil from shipwrecks—if it is heavy fuel—settles on the seabed and some fractions will be present in the sediments. This endangers marine flora and fauna, disrupting the marine ecosystem’s proper functioning^32–35,22^.

In light of the above statements, an important question arises how to test the penetration of oil substances from the wreck into the sediments and seawater? Methods for detecting and eliminating oil spills on the surface are widely developed, including both radar and satellite methods^36–41^. Research studies show that satellite coverage does not necessarily cover the entire extent of an oil spill. In situ observations and oil spill transport modelling were used to examine the full extent of the spill^42^. During the oil spill caused by the Deepwater Horizon explosion in 2010, a wide spectrum of methods were used to detect, identify and eliminate oil^43^. Moreover, in situ techniques, such as the widely used optical fluorosensor, were applied^44–50^. Oil leaks appearing in the seawater column and originating from objects lying on the seabed are relatively little known and require observation for possible threats to the marine environment.

To detect oil substances on the seabed, visual assessment is used, in which divers and remotely operated vehicles are involved^51–52^. Moreover, based on the material from the collected bottom sediments, chemical analyses are carried out using mass spectrometry or fluorometry^53–55^. Acoustic methods, using echo sounders, are considered useful for determining the extent of contamination^56–58,22^. The presented methods refer to examining sediments in search of oil substances. However, at this point, the next question arises, i.e. do fractions of oils present in sediments get into the seawater? Therefore, the purpose of the work is to establish whether the oil present in the sediments penetrates the seawater.

To answer this question, analyses of near-seabed water and sediment samples collected from the area surrounding the wreck site were conducted. It is taken into account that the chromophores found in the structures of PAHs make these compounds fluorescently active when excited by ultraviolet light^59–62^. Therefore, the presence of photoluminescent active hydrocarbons in sediments was assessed. For the analysis of the bottom seawater samples and sediments, excitation-emission spectroscopy (EEMS) was used. Based on the excitation-emission spectra (EEMs), we determined the wavelength-independent fluorescence maximum parameter, which describes the fluorescence maximum by the excitation wavelength corresponding to the emission wavelength. However, to confirm oil presence in the samples, the Fluorometric Index (FI) was developed. Based on the EEMs, the calculations of the Fluorometric Index (FI) were performed. A comparative analysis of sediments and bottom seawater in terms of fluorescent properties, the determined fluorescence maxima and the Fluorometric Index for individual samples allowed for drawing conclusions about the presence of toxic petroleum substances in the sediments and their penetration into the bottom seawater.

## Materials and methods

### Research area

The sampling area is located near the *s/s Stuttgart* wreck site, which is at the bottom of the Baltic Sea, in the Gulf of Gdańsk near the Port of Gdynia in Poland (map position 54°33′33.7″ N, 18°37′1.9″ E). In terms of ecological importance, the area surrounding the *s/s Stuttgart* shipwreck site is the most valuable region in the Polish waters of the southern Baltic. The natural values of the area are reflected in the numerous forms of nature and landscape protection established there, including the Coastal Landscape Park, nature reserves, and *Natura 2000* sites.

The remains of the ship are located at a depth of 21 m. Figure 1 shows a map with six sampling points being marked, where water and sediment samples were collected, and hydrophysical parameters were measured. The location of individual measurement stations along with the sampling depth are presented in Table 1. The choice of sampling points is dictated by the wreck’s location and the seafloor topography in the area, which affects the movement of sediments that shift gravitationally towards deeper regions. Five survey points (2–6) were planned along the line of the descending bottom, starting in the vicinity of the wreck. One point (1) was chosen to be situated in the opposite direction (0.5 km from the wreck), intended as a reference point.

### Research material

The seawater samples from the near-seabed water layers as well as sediment samples for six sampling points (depths in Table 1) were collected using a batometer. The water was transported in glass bottles to the laboratory for further analysis.

**Table 1:** The locations of the sampling points and the sampling depths.

Sampling point (according to the map in Fig. 1)	Position	Sea depth [m]	Water sampling depth [m]	Distance from the centre of the wreck [km]
1	18°36′35″ E 54°33′22″ N	18.49	18	0.50
2	18°36′57″ E 54°33′32″ N	19.83	18.5	0.08
3	18°37′10″ E 54°33′36″ N	22.87	22	0.19
4	18°37′13″ E 54°33′39″ N	23.30	22.5	0.29
5	54°33′39″ N 54°33′44″ N	23.69	23	0.50
6	18°37′32″ E 54°33′47″ N	24.63	24	0.71

#### Seawater

**Fig. 2:**
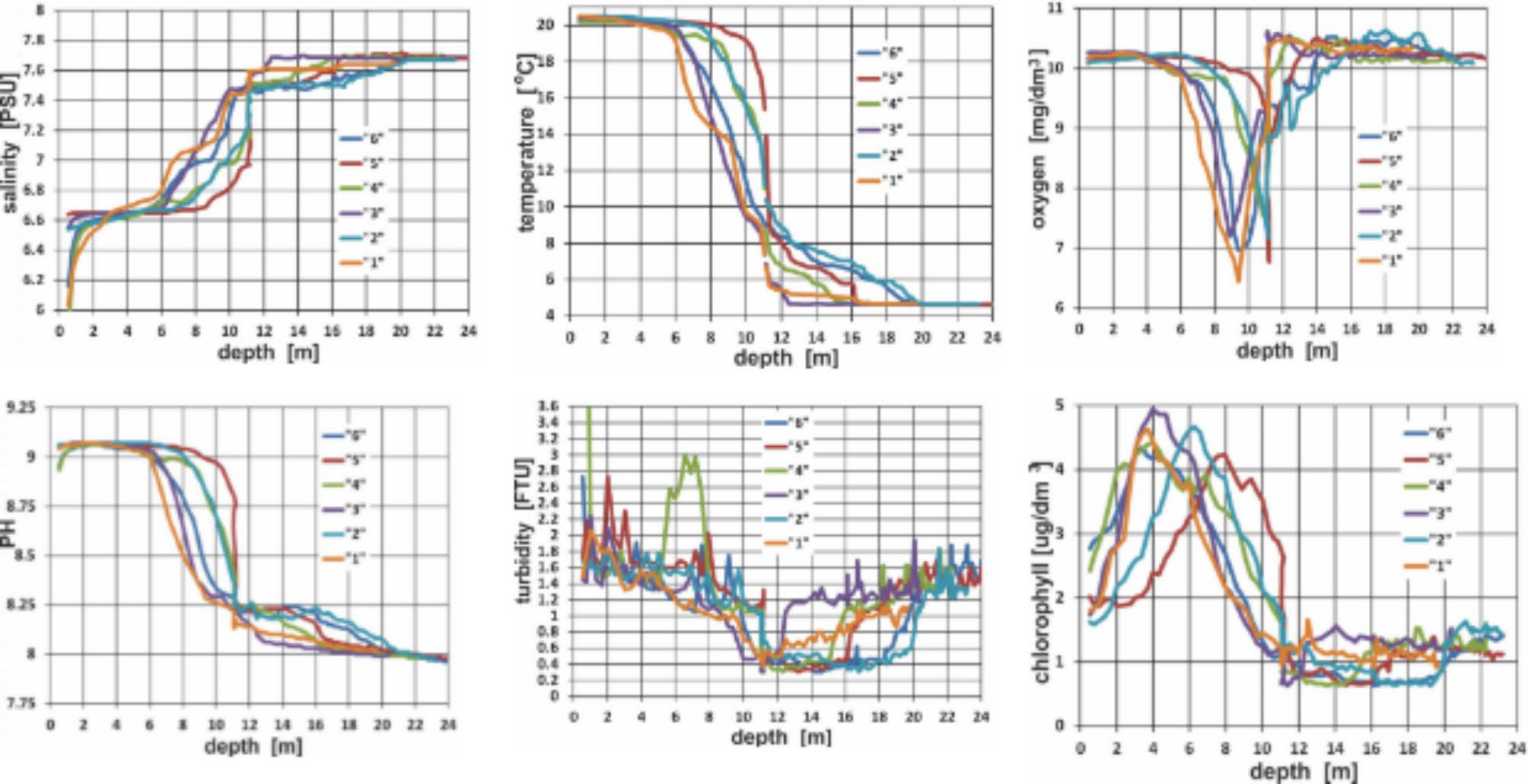
Selected seawater parameters as a function of depth for the considered sampling points.

Since the influence of hydrophysical conditions on the relationships between the presence of oil substances in sediments and bottom seawater cannot be ruled out, water parameters were determined in the water column. Measurements were taken using a multi-parameter CTD 115 M probe by Sea & Sun Technology GmbH (Trappenkamp, Germany) from aboard the motorboat IMOROS 2 (Maritime Institute of the Gdynia Maritime University) on June 23 and 24, 2023. Bottom sediments were collected immediately after collecting the water samples and multi-parameter profiling. The device is equipped with sensors for oceanographic measurements of physical, chemical, and optical parameters. When the instrument was lowered to the bottom, data were recorded every second with a constant sampling frequency (approximately eight measurements per metre). The following parameters were measured: salinity, temperature, concentration of molecular oxygen, pH, turbidity, and chlorophyll concentration. The results are presented as a function of depth in Fig. 2.

The analysis of the results for near-seabed water sampling in the range of the considered depths from 18 to 24 m shows that salinity changes slightly from 7.61 to 7.69, and temperature changes slightly from about 5 °C for a depth of 18 m (point no. 1) to 4.60 °C for 24 m (point no. 6). The concentration of molecular oxygen availability decreases slightly with depth (18–24 m) from 10.40 to 10.16 mg/L. However, pH increases with depth (in the range from 18 to 24 m) from 7.61 to 7.98, and turbidity also increases from 0.4 FTU to 1.6 FTU. Chlorophyll concentration is constant in the considered depth (18–24 m) and the mean chlorophyll is equal to about 1 µg/dm^3^.

#### Sediments

Surface sediment sampling was conducted using a Van Veen grab. During sample collection, the grab was attached to a steel rope fixed to a hydraulic lift and lowered from the side of the vessel to the seabed. The grabbing surface of the sampler is 0.1 m^2^, and it weighs approximately 6 kg. The sampler allows for collecting sediment samples directly from the seabed to a depth of 10–20 cm, depending on local soil conditions. On board the vessel, the grab was opened, and the samples were placed and stored in plastic containers (zip-lock pouches) designed for this purpose.

**Fig. 3:**
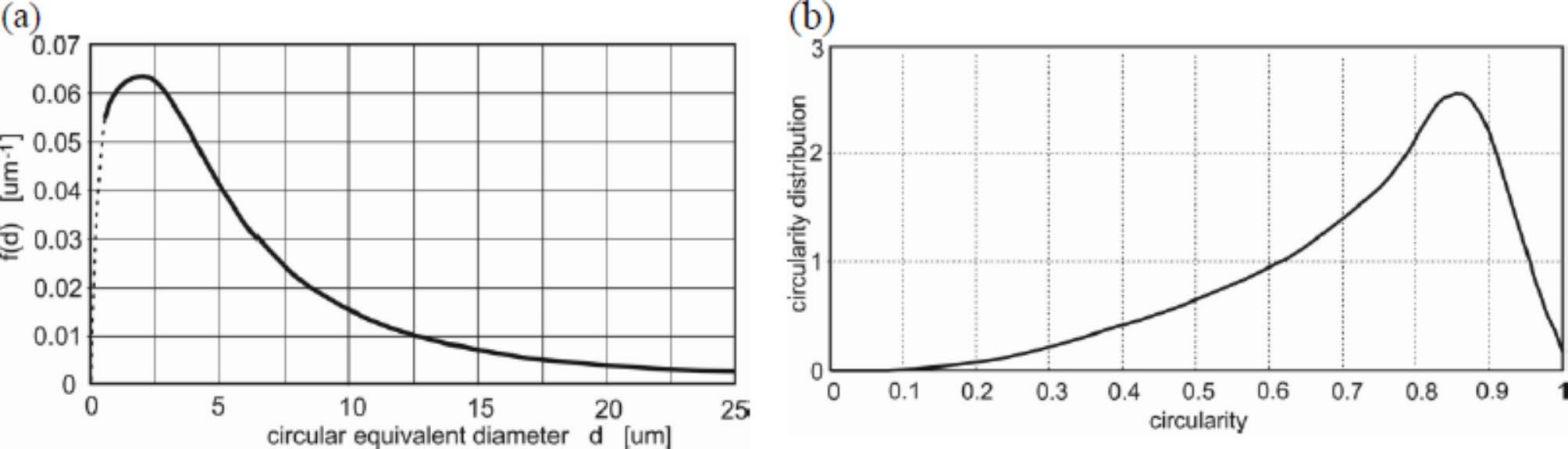
The size distribution of the sediment grains (**a**); the circularity distribution of the grains (**b**).

Physical properties of the dried sediment samples, i.e. sediment size distribution, circularity and elongation were determined. For this purpose, measurements were carried out using an automated particle analyser (*Morphologi G3*), which enables simultaneous determination of the shape and size of every grain. One million grains of sediments were analysed. The obtained parameters for sediment characterisation are presented in Fig. 3. Figure 3a shows the size distribution of all grains from sampling point no. 2. The integral for values from 0 to the diameter size of the largest grain is 1, which mathematically means that a given grain has a 100% probability that its diameter ranges from 0 to the maximum diameter size (the integral over a given range of diameters determines the probability that the particle diameter is contained in this range). The average diameter is 6.6 μm, 50% of the grains have a diameter greater than 4.9 μm, 20%—greater than 8.9 μm, 10%—greater than 13.7 μm, and 1%—greater than 42 μm. Figure 3b presents the circularity distribution of all grains. The integral for values between 0 and 1 is 1, which mathematically means that a given grain has a 100% probability that its circularity ranges from 0 to 1. A circularity value of 1 means that the grain image does not deviate from the shape of a circle, while a value of 0 means that the shape deviates as much as possible from the shape of a circle (the instrument interprets the shape as linear). Sediments from other points do not differ noticeably.

**Fig. 4:**
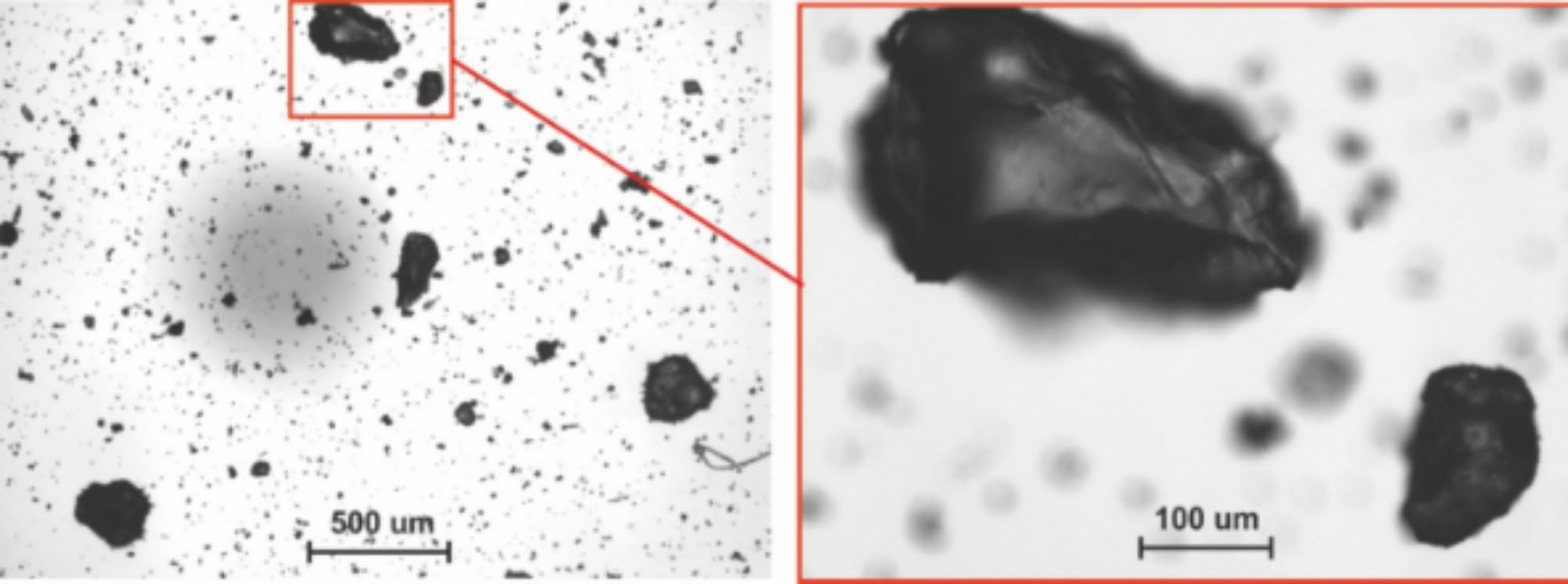
A sample photo of dried sediment (from an automated particle analyser).

Figure 4 presents photos showing the grains of sediments, which were obtained using a particle analyser *Morphologi G3*.

### Fluorescence apparatus

Fluorescence measurements based on excitation-emission spectra (EEMs) both for the bottom seawater samples and sediment samples were performed.

The measurements of EEMs of the sediment samples were performed in artificial seawater with 8 PSU. The initial sediment concentration was prepared by adding 20 ml of sediments to 60 ml of artificial seawater. Next, based on the dilution method, the starting sediment concentration in seawater was diluted four times and used for EEM measurements. As a standard, MiliQ ultrapure water was used.

To determine the EEMs of the seawater samples and sediment samples after contact with water, a Hitachi F-7100 FL spectrofluorometer was used. The measurements of EEMs of the samples were performed using a 1 × 1 cm quartz cuvette at a constant temperature of about 20 °C. During the EEM measurements, the excitation wavelength was changed from 200 to 480 nm with an excitation wavelength interval of 5 nm; the emission wavelength was changed from 260 to 600 nm with a 5 nm emission interval, a 10 nm excitation slit, and a 10 nm emission slit. The integration time was 0.5 s, and the photomultiplier tube voltage was 400 V.

## Results

To answer the study’s main objective, the analysis of the sediment samples and the bottom seawater samples was performed in two parts. In the first part, based on the measurements, excitation-emission spectra (EEMs) were determined to characterise the fluorescence properties of the samples. Next, based on the determined EEM matrices of oil emulsions, the following spectral signatures were analysed: changes in the wavelength-independent fluorescence maxima occurring in the EEMs, and changes in the shape of fluorescence intensity in relation to the individual station under consideration. In the second part, the calculations of the oil index, the so-called Fluorometric Index (FI), were performed based on the determined EEMs.

### Excitation-emission spectra (EEMs) of the samples

Oil substances exhibit fluorescent properties caused by aromatic hydrocarbons present in their structure, which manifest their presence with a characteristic fluorescence spectrum when excited by light of a specific wavelength. In this work, a method based on excitation-emission spectra (EEMs), which provides a set of excitation wavelengths and the corresponding emission wavelengths, was used to detect oil substances in the samples. This allows for easily determining the characteristic fluorescence maxima of fluorescing components in the tested samples. Each of fluorescence maxima in the EEM spectrum has been described by the wavelength-independent fluorescence maximum (λ_Ex_/λ_Em_)^63^ in relation to fluorescing substances naturally present in seawater, which exhibits its own characteristic fluorescence spectrum. This is due to the organic matter present in the water, i.e. coloured organic matter (CDOM) and humic acids. The wavelength-independent fluorescence maximum parameter data (λ_Ex_/λ_E**m**_)^63–64,66^ are presented in Table 2. Research on the detection of oil contaminants in seawater based on EEM spectra was conducted by Baszanowska and Otremba^59–62,65^. As part of a series of procedures, the wavelength-independent fluorescence maximum (λ_Ex_/λ_Em_) parameters characteristic of oil substances in seawater were determined^61^ and they are presented in Table 3. These studies were carried out in relation to the oil fraction dissolved in near-surface water, collected at a depth of 1 m.

**Table 2:** Major fluorescent components of seawater with their wavelength-independent fluorescence maxima (λ_Ex_/λ_Em_)^63–64,66^.

Seawater component	Peak name	Ex_max_ [nm] / Em_max_ [nm]
Tyrosine-like	B	225–237/309–321, 275/305–310
Tryptophan-like	T	225/340–390, 275/320–350
UVC-humic-like	C	300–370/380–480, 320–360/420–460
UVA-humic-like	A	247–260/380–500, 260/400–460
Marine humic-like	M	290–310/370–410
Pigment-like	P	398/660

**Table 3:** Major fluorescent peaks of oil substances in natural seawater at oil-to-water ratios of 0.5–500 × 10^− 6^, with their wavelength-independent fluorescence maxima (λ_Ex_/λ_Em_)^61^.

Ex_max_ (nm) ± 5 (nm) / Em_max_ (nm) ± 5 (nm)				
Oil substance	Peak 1	Peak 2	Peak 3	Peak 4
Crude oil, lubricating oil, fuel	220–225 / 330–340	215–225 / 295–310	250–255/360	200–275 / 310–335

Based on the collected literature data regarding the fluorescence maxima of the parameter (λ_Ex_/λ_Em_), the determined EEM spectra for sediments dissolved in water and the bottom waters were analysed.

### EEMs of sediments

**Fig. 5:**
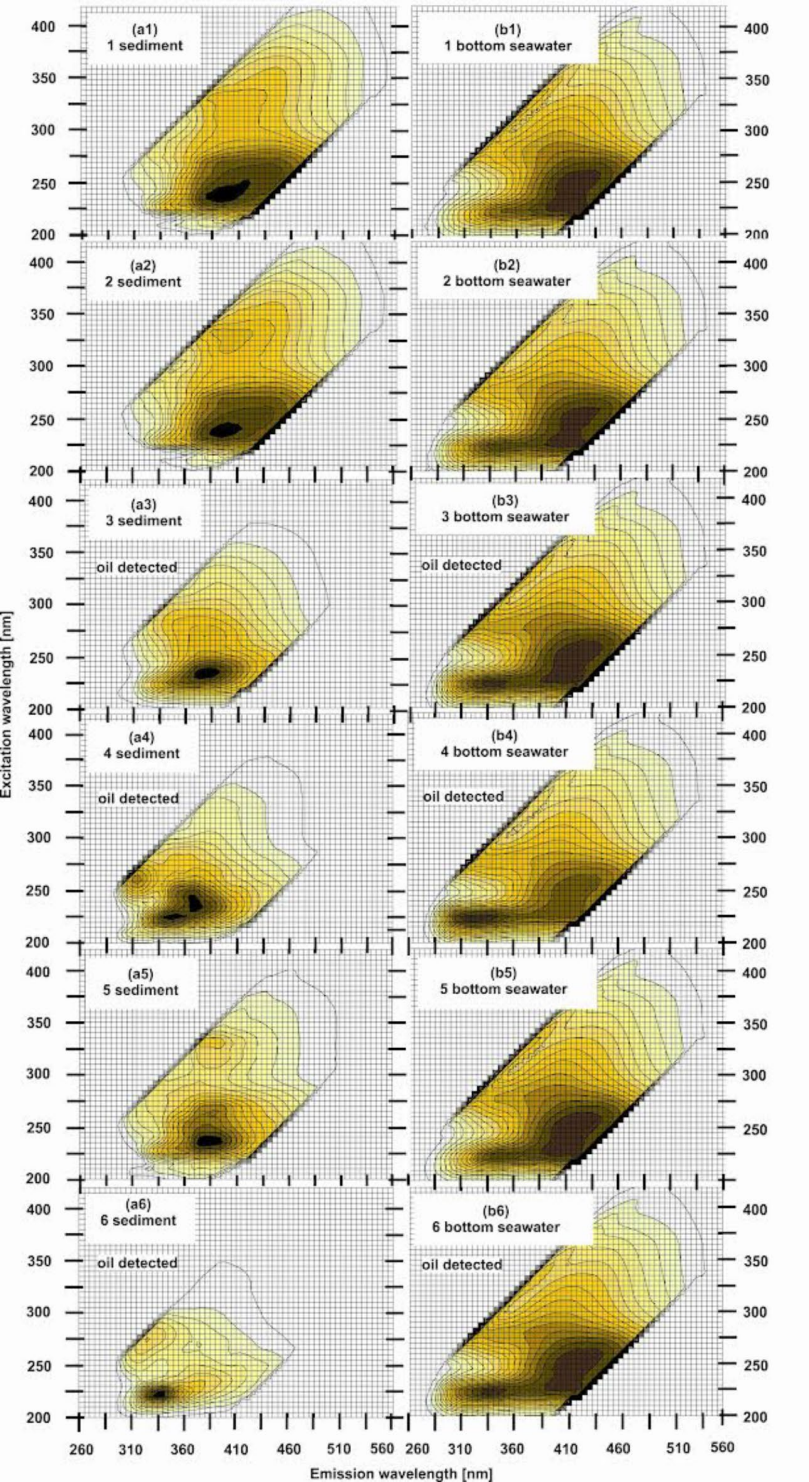
EEMs for various sampling points (no. 1–6) for the sediments (**a1**–**a6**) and seawater collected from the bottom (**b1**–**b6**) near the *s/s Stuttgart* shipwreck in the Gulf of Gdańsk. EEMs ​​corresponding to oil-contaminated sediments are marked with “oil detected” (**a3**, **a4** and **a6**).

Figure 5(a1–a6) shows the sediment EEMs in 2D for individual sampling points no. 1–6. The characteristic fluorescence maxima described by the wavelength-independent fluorescence maxima (λ_Ex_/λ_Em_) for individual sampling points no. 1–6 for the sediment samples were determined (see Tables 2 and 3):

- (1) 230/355 (T), 235/390 (T), 330/390, 330/415 (hum.-like C),
- (2) 225/360 (T), 235/400 (hum.-like A ) and 310/400, 320/395 and 330/410 (hum.-like C),
- (3) 225/350 (oil peak see Table 3 ), 230/385 (T), 275/380 (hum.-like C),
- (4) 225/350 (oil peak see Table 3), 240/375 (T) and 260/315 (oil peak see Table 3),
- (5) 225/355 (T), 225/375 (T), 275/390 (hum.-like A), 335/385 (hum.-like C),
- (6) 220/340 (oil peak see Table 3) and 275/325 (oil peak or T).

Table 4 shows the fluorescence maxima (λ_Ex_/λ_Em_) determined for the sediment samples. Based on the peak analysis of the sediment samples for individual sampling points, we can conclude that oily substances are present in the sediment samples from sampling points no. 3, 4 and 6. However, the determined peak (λ_Ex_/λ_Em_) = 225/350 is controversial because it does not indicate the presence of oil; it may also be responsible for the presence of tryptophan—a component of CDOM. Therefore, for point no. 3, it cannot be clearly stated that oil is present in the sediment.

**Table 4:** Major fluorescent peaks with their wavelength-independent fluorescence maxima (λ_Ex_ / λ_Em_) for sediments for sampling points no. 1–6 near the *s/s Stuttgart* shipwreck in the Gulf of Gdańsk.

Ex_max_ [nm] ± 5 [nm]/Em_max_[nm] ± 5 [nm]				
Sampling point	Peak (T)	Peak (A)	Peak (C)	Oil peak
1	230/355 235/390		330/415 330/390	
2	225/360 235/400		325/395 330/410 310/400	
3	225/350 235/385	275/380		225/350
4	225/350 240/375			225/350 260/315
5	225/355 225/375	275/390	335/385	
6	230/380 275/325			220/335

### EEMs of bottom seawater

Figure 5 (b1–b6) shows the bottom seawater EEMs in 2D for individual sampling points no. 1–6. The particular fluorescence maxima (λ_Ex_/λ_Em_) for individual sampling points (no. 1–6) for the bottom seawater were determined (see Tables 2 and 3):

- (1) 225/360 (T), 235/405 (hum.-like C), 255/415 (hum.-like A), 280/380 (M), 295/405 (M),
- (2) 225/250 (T), 230/400 (hum.-like C), 250/420 (hum.-like A), 275/350 (T), 295/405 (M),
- (3) 225/340 (oil see Table 3), 230/400 (hum.-like C), 255/415 (hum.-like A), 280/375 (M), 295/405 (M),
- (4) 225/325 (oil see Table 3), 225/405 (hum.-like C), 260/425 (hum.-like A), 295/405 (M),
- (5) 225/350 (T), 255/415 (hum.-like A), 295/405 (M), 275/350 (T), 295/405 (M),
- (6) 225/340 (oil see Table 3), 255/420 (hum.-like A), 295/405 (M), 280/375 (M).

The determined peaks (λ_Ex_/λ_Em_) = 225/340 and 225/325 indicate the presence of oily substances (see Table 3). The peaks were determined for sampling points no. 3, 4 and 6. On this basis, it can be concluded that polycyclic aromatic hydrocarbons are present in the bottom water samples collected at points no. 3, 4 and 6. Table 5 shows the determined fluorescence maxima (λ_Ex_/λ_Em_) for the bottom water samples.

**Table 5:** Major fluorescent peaks with their wavelength-independent fluorescence maxima (λ_Ex_ / λ_Em_) for the bottom seawater for sampling points no. 1–6 near the *s/s Stuttgart* shipwreck in the Gulf of Gdańsk.

Ex_max_ [nm] ± 5 [nm]/Em_max_[nm] ± 5 [nm]					
Sampling point	Peak (T)	Peak (A)	Peak (M)	Peak (C)	Oil peak
1	225/360	255/415	295/405 280/380	235/405	
2	225/350 275/350	250/420	295/405	230/400	
3		255/415	295/405 280/375	230/400	225/340
4		260/425	295/405	225/405	225/325
5	225/345 275/350	255/415	295/405		
6		255/420	295/405 280/375		225/340

### Fluorescence intensity of the samples

**Fig. 6:**
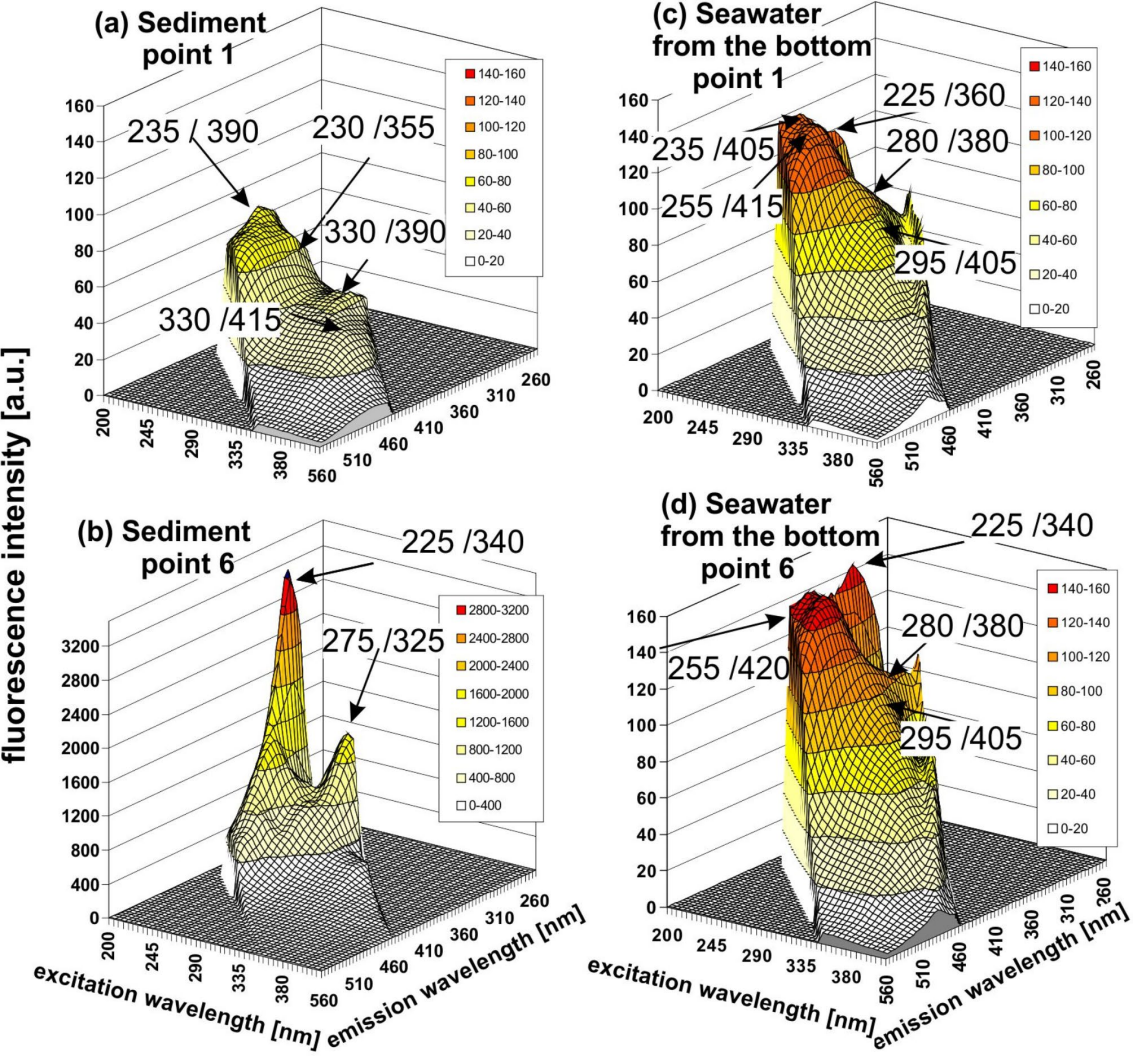
The EEMs in 3D for sediments [point no. 1(a) and point no. 6 (b)] and for bottom seawater [point no. 1(c) and point no. 6 (d)] near the *s/s Stuttgart* shipwreck in the Gulf of Gdańsk. In the figure, particular determined (λ_Ex_/λ_Em_) parameters were assigned. The fluorescence maximum (λ_Ex_/λ_Em_) = 225/340 determined for point no. 6 is responsible for the oil presence.

Moreover, the fluorescence intensity of the peaks (λ_Ex_/λ_Em_) detected in the EEM spectra both for the sediment and bottom water samples was analysed. Figure 6 shows the EEMs in 3D for two selected sampling points: point no. 1, as a representative of the stations with no oil substances being detected (see Tables 4 and 5), and point no. 6, as a representative of stations where oil substances were detected. In Fig. 6, characteristic detected peaks (λ_Ex_/λ_Em_) were marked. In the sediment EEM spectrum for point no. 6, the highest fluorescence intensity value of about 3400 for (λ_Ex_/λ_Em_) = 225/340 was determined. As it was mentioned above, the (λ_Ex_/λ_Em_) = 225/340 is responsible for the oil presence. In the case of the bottom water samples from point no. 6, the fluorescence intensity values ​​are much lower. However, the highest fluorescence intensity value of approximately 150 was recorded for (λ_Ex_/λ_Em_) = 225/340, which is responsible for the oil presence. In the case of station no. 1, the determined fluorescence intensity value ​​for the sediment sample is approximately 70 for (λ_Ex_/λ_Em_) = 235/390 (T), while for the bottom water sample, the highest fluorescence intensity value is approximately 120 for (λ_Ex_/λ_Em_) = 235/405 (C).

### Fluorometric index definition

To confirm the oil presence in the sediment samples and bottom seawater samples the calculations based on an oil index – the Fluorometric Index (FI) were performed. The FI was defined by Baszanowska and Otremba^61–62^ as the quotient of the fluorescence intensity of oil-polluted seawater at the emission wavelength to the fluorescence intensity of oil-free seawater at the same maximum excitation wavelength for both polluted and oil-free seawater (see Formula 1). Calculations of the FI were performed based on Formula 2, where the intensity of oil-polluted seawater (I(λ_Em_ of oil-polluted seawater)) is divided by the intensity of oil-free seawater (I(λ_Em_ of oil-free seawater)) at 340/355 for λ_Ex_ = 225 nm.

1 $$F{I_{{o \mathord{\left/{\vphantom {o w}} \right.\kern-\nulldelimiterspace} w}}} = {\left[ {\frac{{I({\lambda _{Emission\;{\kern 1pt} of\;seawater\;polluted\;by\;oil}})}}{{I({\lambda _{Emission\;{\kern 1pt} of\;natural(oil - free)\;seawater}})}}} \right]_{{\lambda _{Excitation}}}}$$

2 $$F{I_{{o \mathord{\left/{\vphantom {o w}} \right.\kern-\nulldelimiterspace} w}}} = {\left[ {\frac{{I\left( {{\lambda _{Em = 340}}} \right)}}{{I\left( {{\lambda _{Em = 355}}} \right)}}} \right]_{{\lambda _{Ex = 225}}}}$$

FI_o/w_ achieved values above 1 when oil substances were present in the sample, while FI_o/w_ achieved values below 1 when the sample was oil-free. The cases when FI_o/w_ achieved values equal to 1 were inconclusive.

### The fluorometric index for the sediment and bottom seawater calculations

The calculated FI values ​​for individual stations for the sediments and bottom water are presented in Table 6. In the case of the sediments, FI values ​​higher than 1 were determined for sampling point no. 3 (1.010) and point no. 6 (1.386). This allows us to conclude that the sediments at these points contain oily substances. However, lower FI values ​​determined for point no. 1 (0.856), point no. 2 (0.842) and point no. 5 (0.900) indicate that the sediment samples are free of oily substances. In the case of the bottom water, FI values ​​higher than 1 were determined for sampling point no. 3 (1.075), point no. 4 (1.101) and point no. 6 (1.064). These results indicate that oil is present in the water and is seeping into the water from sediments. However, the FI achieved values ​​lower than 1 for the bottom water in the case of point no. 1 (0.941) and 5 (0.998), similar to the case of the sediments. An inconclusive FI result was assigned to point no. 4 (1.000) for the sediments. This result does not indicate whether oil is present or not. However, peak (λ_Ex_/λ_Em_) = 260/315 determined from the EEMs for sampling point no. 4 indicates the presence of oil in the sample^61^.

The FI enables dispelling doubts regarding the presence of oil in sediments for sampling point no. 3, where FI value = 1.010. This allows us to conclude that oily substances are present in the sediments at point no. 3. This could not be clearly stated in the case of the EEM peak analysis for this station.

**Table 6:** The calculated *FI*_o/w_ values for the sediments and bottom seawater for various sampling points.

FI_o/w_ [–]		
Sampling point no.	Sediments	Bottom seawater
1	0.856	0.941
2	0.842	1.000
3	1.010	1.075
4	1.000	1.101
5	0.900	0.998
6	1.386	1.064

## Discussion

The conclusion about the presence of oil was based on three premises related to fluorometric spectra. These are increased fluorescence intensity, the occurrence of peaks characteristic of oily substances, and an increased Fluorescence Index (FI) value, which were identified for the sediments from three sampling points (no. 3, 4 and 6). As for the bottom water, the samples showed no significant differences in fluorescence intensity, but oil peaks emerged, similarly to increased FI values. In the applied method, the presence of oil in the sediment is signaled by the presence of substances alien to a given environment, i.e. aromatic hydrocarbons. The proposed fluorometric method enables the detection of oil. Unfortunately, we are not able to determine the type of individual chemical compounds and the concentration of oil substances. In addition, patches of heavy fuel oil may be located next to sediment collection site. We “see” substances which are released from the oil and diffuse in the mass of the sediment or pass into the bottom water.

No oil was found at three sediment and bottom water sampling points: no. 1 (reference point), 2 and 5. It is, therefore, very likely that oil disturbs the seabed unevenly. It is possible that the oil was released from the leaking tank because it was gradually being pushed out by gravity by the water. Probably, after the oil was released from the tanks, it did not melt at the bottom but floated to the water surface, where it underwent weathering processes. When the density of the oil began to exceed the density of the water, the oil began to sink, and a film created by hydrocarbons leached from heavy fuel oil (HFO) remained on the surface for some time. It is also possible that the oil was thrown into the water as a result of explosions caused to dislodge parts of the ship that was an obstacle to navigation. While in the water, the oil could have been moved in the direction of sea currents before settling at the bottom. It is also possible that the oil flowed directly to the bottom surface and did not spread evenly (the influence of bottom currents or unevenness of the bottom).

Disputable is what methodology should be used to determine the presence or content of hydrocarbons. This concerns both the method and frequency of sampling and the methods of laboratory analysis. Typical analytical methods for estimating hydrocarbons include gravimetry (non-volatile extractables), UV and IR absorption, gas chromatography, and gas chromatography/mass spectrometry. However, the above-mentioned methods require complex laboratory activities and take a lot of time. The process of obtaining oil substances from water (extraction) requires a large volume of samples.

In the case of the method now used, it is necessary to collect samples from the environment and perform a fluorometric analysis in the laboratory. In terms of volume, these samples are small, around 100 mL. They also do not require laboratory processing before being placed in a spectrofluorometer. The Fluorometric Index used in this work was derived from the three-dimensional fluorescence spectra. This parameter uses one excitation wavelength and two emission wavelengths. Therefore, it is unnecessary to determine the full excitation-emission matrix (EEM). This provides a premise for constructing a simplified fluorometric device for detecting oil substances that could work as underwater sensors. The fluorometric method manifests hydrocarbon compounds containing fluorophores in their structure, such as cyclic and polycyclic hydrocarbons (PAHs), widely considered harmful (toxic, carcinogenic, mutagenic).

**Fig. 7:**
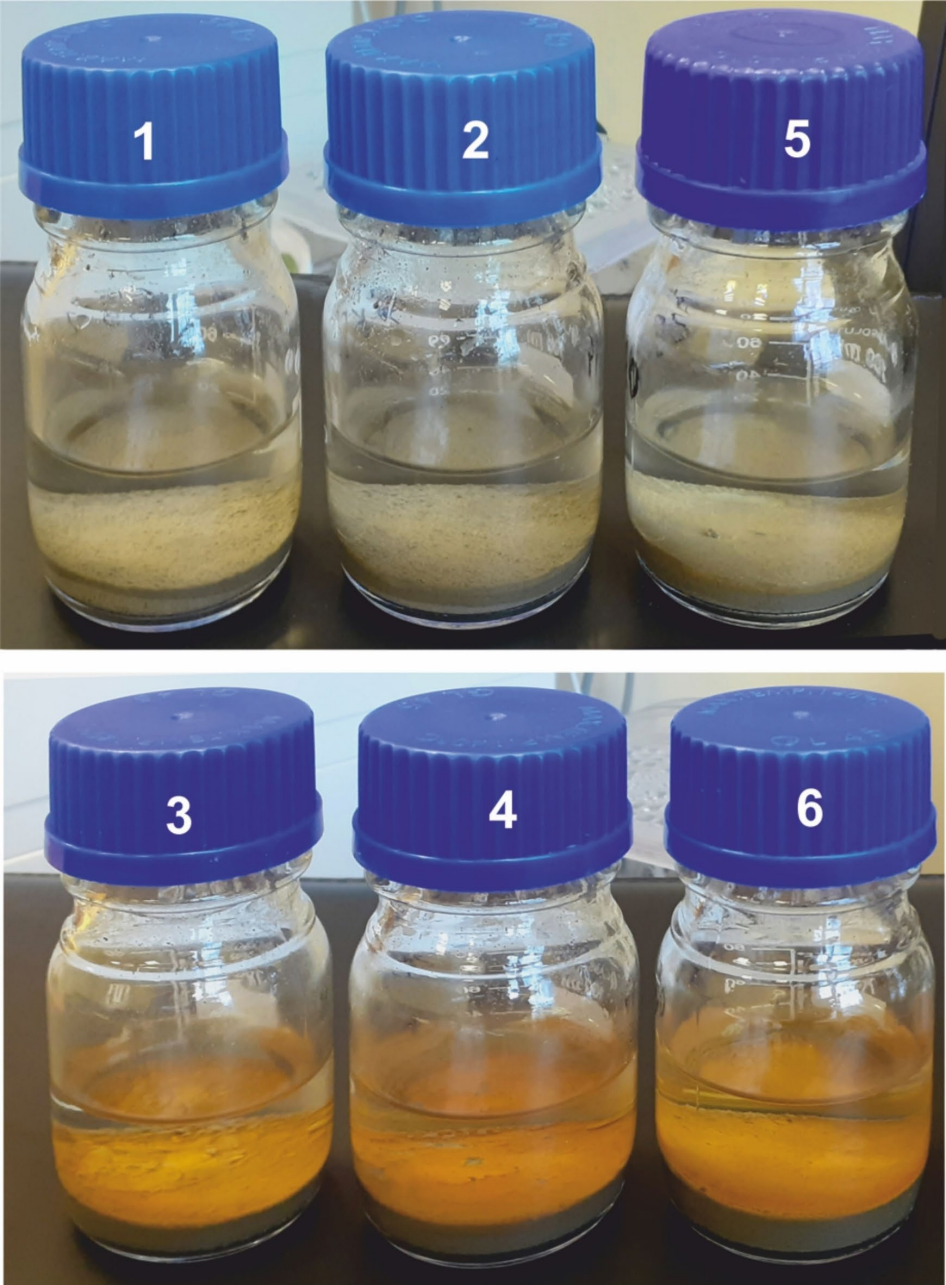
Sediment appearance (1, 2 and 5 identified as oil-free, and 3, 4 and 6 as oil-polluted).

The fluorometric method does not allow for determining the content of aliphatic hydrocarbons, but they do not cause as many environmental problems as aromatic hydrocarbons. Aliphatic hydrocarbons predominate in the composition of crude oil. They may also probably affect some components of the environment. Some bacteria develop their enzymatic apparatus towards oily substances, thus taking part in the remediation of the oily environment. In the case of the research described, additional observations were obtained that may be related to oil in marine sediments, i.e. a change in the surface colour of the sediment which is considered to be contaminated with oil. An orange-red coating (Fig. 7) appears after several days of storage at room temperature.

The nature of this phenomenon has not yet been determined. One can only assume that the colour change may be related to the interaction between oil present and bacterial activity. It is worth conducting a deeper analysis of this phenomenon in the future.

Above, in the “Research and Material” section, the physical characteristics of both the sediments and the water above them are presented. However, at the current stage of research, it is not possible to identify possible connections between the behaviour of oil substances originating from the wreck and the physical properties of the sediments and seawater. The grain size characteristics of the sediments are not related to the presence of oil in them. The same applies to the hydrophysical parameters of water—it cannot be stated that their values ​​at the bottom and across the entire water column could affect the detection of oil substances.

## Conclusions

The aim of the study was to answer the question of whether harmful oil-derived substances present in sediments are released into seawater. Therefore, it included an analysis of samples of bottom layers of water and sediments for the content of harmful fluorescent oil-derived substances in ultraviolet. Samples were collected from the site of a WW2 German shipwreck, i.e. the *s/s Stuttgart* shipwreck, which lies in the Baltic Sea, in the Gulf of Gdańsk near the Port of Gdynia. The fluorometric analyses, which were performed in three stages, including the determination of excitation-emission spectra (EEMs), fluorescence intensity and the Fluorometric Index (FI), were to establish whether hydrocarbons present in the wrecks affect the bottom seawater. The fluorometric analyses of the examined seabed sediment samples and bottom water samples in the vicinity of the wreck showed oil pollution of the sea environment at selected measurement sampling points close to the wreck. Oil substances were detected both in the sediments and near-bottom water for three sampling points, which indicates the patchwork location of oil in the sediments. The findings highlight the ongoing risk posed by such wrecks to the marine ecosystem and underscore the need for continuous monitoring and mitigation efforts to protect the natural environment of the Gulf of Gdańsk. The fluorometric method appears to be effective in identifying locations where residual oil substances in sediments are released into the seawater.

## Data Availability

The datasets used and analysed during the current study are available from the corresponding author on reasonable request.
